# Incidence of urological tumors in Down’s syndrome: a systematic review and meta-analysis

**DOI:** 10.1007/s11255-023-03656-4

**Published:** 2023-06-27

**Authors:** Fernando Korkes, Maria Paula Gomez-Bueno, Herney Andrés García-Perdomo

**Affiliations:** 1 Universidad ABC, Santo André, Brazil; 2 UROGIV Research Group, School of Medicine, Universidad del Valle, Cll 4b #36-00, Cali, Colombia; 3 Division of Urology/Urooncology, Department of Surgery, School of Medicine, Universidad del Valle, Cali, Colombia

**Keywords:** Urological tumors, Urological cancer, Down’s syndrome, Trisomy 21, Incidence, Frequency

## Abstract

**Background:**

Some authors have estimated that the incidence of testicular germ cell tumors in individuals with trisomy 21 is more than fivefold higher than that in the general population.

**Objective:**

This systematic review aimed to estimate the incidence of urological tumors in patients with Down’s syndrome.

**Study design:**

We conducted a search strategy in MEDLINE (OVID), EMBASE, LILACS, and the Cochrane Central Register of Controlled Trials (CENTRAL) from inception to nowadays. We assessed the risk of bias and performed a meta-analysis. Also, the heterogeneity between trials was evaluated by the *I*^2^ test. We completed the subgroup analysis based on the type of urological tumor (testis, bladder, kidney, upper urological tract, penile, retroperitoneum).

**Results:**

We found 350 studies by the search strategy. After carefully reviewing, full-text studies were included. 16,248 individuals with Down’s syndrome were included, and 42 patients presented with urological tumors. There was a total incidence of 0.1%, 95%CI (0.06–0.19), *I*^2^ 61%. The most common urological tumor reported was testicular. We found six studies describing 31 events and an overall incidence of 0.19%, 95%CI (0.11–0.33), *I*^2^: 51%. Other studies reported kidney, penile, upper urinary tract, bladder, and retroperitoneum tumors with a very low incidence, 0.02%, 0.06%, 0.03%, 0.11%and 0.07%, respectively.

**Discussion:**

Regarding non-testicular urological tumors, we found incidences as low as 0.02% in kidney cancer or 0.03% in the upper-urothelial tract tumors. It is also lower than the general population. Compared to the age of onset of patients, it is also lower than the general population, perhaps related to a shorter life expectancy. As a limitation, we found a high heterogeneity and a lack of information regarding non-testicular tumors.

**Conclusion:**

There was a very low incidence of urological tumors in people with Down’s syndrome. Testis tumor was the most frequently described in all cohorts and within a normal distribution range.

## Introduction

Down syndrome is a congenital abnormality characterized by the presence of all or part of a third copy of chromosome 21 [1]. It is the most common chromosomal disorder associated with a spectrum of physical and functional disability and a predisposition to developing hematopoietic malignancies, mainly leukemia [1–3]. The solids tumors are less frequent than the general population, except for testicular cancers [3–5]. Some authors have estimated that the incidence of testicular germ cell tumors in individuals with trisomy 21 is more than fivefold higher than in the general population [6]. In line with that, a decreased risk of dying from other urological cancers, such as bladder or kidney, has been seen in persons with Down’s syndrome [7].

In chromosome 21, the Down syndrome critical region (DSCR), an overexpression is responsible for the phenotypic features observed, such as mental retardation and developmental heart defects [8, 9]. Many studies indicate a possible role as a tumor suppressor gene; however, others prove its pro-oncogenic activity [10, 11]. Some hypotheses have been raised that the extracellular matrix secreted by fibroblasts from individuals with DS suppresses the proliferation of stromal-rich tumors (e.g., breast cancer), but not stromal-poor tumors (e.g., lymphoproliferative disorders, retinoblastoma, and germ cell tumors) [12].

Data on the incidence or frequency of solid tumors (specifically urological tumors) in this population are heterogeneous, in contrast to hematopoietic malignancies, where they are widely known [5]. The need to check this information has caught our attention—this systematic review aimed to estimate the incidence of urological tumors in patients with Down’s syndrome.

## Methods

We conducted this systematic review following the Cochrane Collaboration recommendations and PRISMA statement.

### Eligibility criteria

Study designs: We included clinical trials and observational studies.

Participants: Patients with Down’s syndrome and any urological tumor were included. There were no preferences in any other demographic characteristic of participants.

Outcomes: The primary outcome was the incidence of urological tumors in patients with Down’s syndrome.

Exclusion criteria: Studies included fewer than five patients with Down’s syndrome and any urological tumor.

### Information sources and search strategy

We designed a search strategy in MEDLINE (OVID), EMBASE, LILACS, and the Cochrane Central Register of Controlled Trials (CENTRAL) from inception to nowadays (Appendix 1). The search strategy was specific for each database and included a combination of the medical subject headings for incidence and Down’s syndrome. To ensure literature saturation, other electronic sources were used to find additional studies’ references from relevant articles identified through the search, conferences, thesis databases, Open Grey, Google Scholar, and clinicaltrials.gov, among others. If some information was missing, we contacted the authors by e-mail in case of missing data. No language or setting restrictions were imposed.

### Data collection

Two researchers (F.K, MPG) reviewed each reference by title and abstract. Then, reviewers confirmed all data in full texts of relevant studies, applied pre-specified inclusion and exclusion criteria, and extracted the data. Disagreements were resolved by consensus, and where disagreement could not be solved, a third reviewer (H.A.G) dissolved the conflict.

Relevant data were collected using a standardized form to extract the following information from each article: study design, geographic location, authors’ names, title, objectives, inclusion and exclusion criteria, number of patients included, losses to follow-up, timing, definitions of outcomes, outcomes, and association measures and funding source.

### Risk of bias

We assessed the risk of bias by the STROBE statement.

### Data analysis/synthesis of results

We performed the analyses in R. We performed a meta-analysis of proportions with the command metaprop and the method inverse (logit transformed proportions). We performed this statistical approach and the subgroup analysis according to the high clinical heterogeneity expected and a vast proportion of variation among studies. We assessed the heterogeneity through the *I*^2^ test, and the values of < 50% and > 50% in the *I*^2^ test represent low and high levels of heterogeneity, respectively. We reported the results as forest plots with a 95% confidence interval (95% CI).

### Sensitivity analysis

We analyzed the sensitivity by extracting weighted studies and running the estimated effect to find differences.

### Subgroup analysis

We performed the subgroup analysis based on the type of urological tumor (testis, bladder, kidney, upper urological tract, penile, retroperitoneum).

## Results

### Study selection

**Fig. 1:**
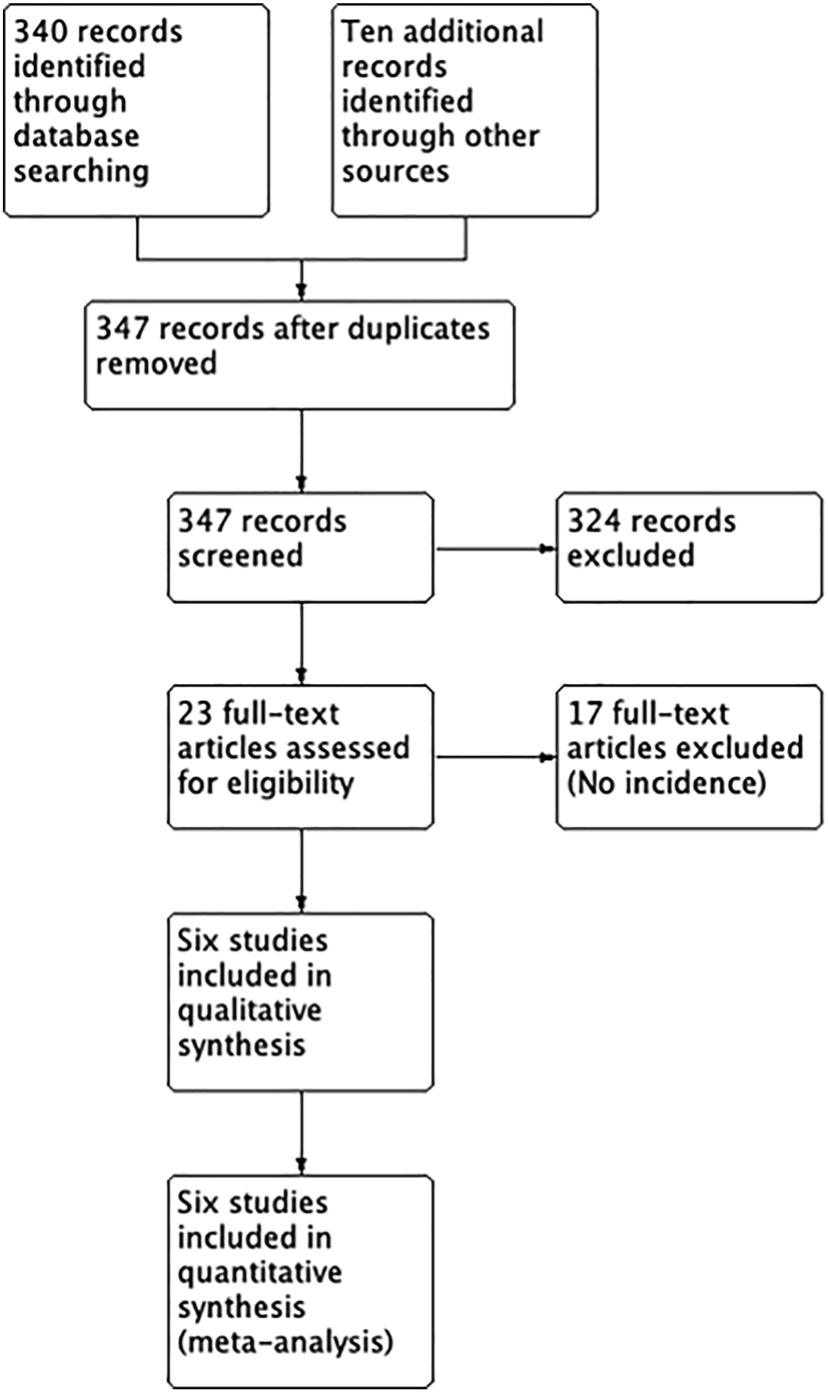
PRISMA flowchart

We found 350 studies by the search strategy. After carefully reviewing full-text studies, we finally included six studies (Fig. 1) [2, 4, 13–16].

### Characteristics of included studies

We found a total number of 16,248 individuals with Down syndrome. The proportion of males and females was not described in all studies. Primary studies came from Sweden, Denmark, Japan, Australia, Finland, and Britain, and they collected information between 1963 and 2012. All studies were retrospective and multi-center, of which five of six were cohorts, and one was case–control. Furthermore, Hill’s (2003) study was based on cohorts and databases, while all the others considered databases and autopsy data.

The age of presentation of those tumors ranged from 15 weeks to 45 years (Table 1).

**Table 1:** Characteristics of included studies

Study	Patients	Years of collection	Study design/methods	Sex	Age	Tumor localization	Urological tumor	Incident rates of SIR (95% CI)
Hill, 2003 [13]	4872	1965 to 1989	Cohort/multi-center. Retrospective. Combined cohort and database	2622 males and 2250 females	Only kidney cancer at age 1 year, others solid tumors > 20 years	Testis 4, penile 3, kidney 1	8	Testis 3.7 (95% CI 1.0–9.4), penile 45.5 (95% CI 9.3–132.8), and kidney 0.6 (95% CI 0.1–3.4)
Hasle, 2016 [4]	3530	1968 to 2012	Cohort/multi-center. Retrospective. Database	1928 males and 1602 females	Median age 35 years, range 18–45 years	Testis 14, pelvis and ureter 1, bladder 4	19	Testis 2.9 (95% CI 1.6–4.8), pelvis and ureter 2.36 (95% CI 0.03–13-1), bladder 1.00 (95% CI 0.27–2.57)
Ehara, 2011 [14]	1514	1974 to 2000	Cohot/multi-center. Retrospective. Autopsy data	831 males and 674 females	Neonate to 64 years	Testis 3, retroperitoneal 1	4	Not available
Sullivan, 2007 [16]	1298	1982 to 2001	Cohort/multi-center. Retrospective. Database	725 males and 573 females	28 years	Testis 1	1	1.94 (95% CI 0.05–10.3)
Patja, 2006 [2]	3581	1978 to 1986	Cohort/multi-center. Retrospective. database	1888 males and 1693 females	Testicular cancer remaining elevated through ages 30–39	Testis 6, kidney 1	7	Testis 4.8 (95% CI 1.8–10.4), kidney 0.5 (95% CI 0.0–2.8)
Goldcare, 2004	1453	1963–1999	Cases and controls/multi-center retrospective database	Not available	Not available	Testis 3	3	Not available

Urological tumors were located in the testicles, penis, kidney, pelvis, ureter, bladder, and retroperitoneum. Only histological subtypes of testicular tumors were described: seminoma, teratocarcinoma, embryonal carcinoma, yolk sac tumor, and mixed germ cell.

### Characteristics of excluded studies

The exclusion criteria were the minimum number of reported individuals, study design, full text unavailable, or no mention of the primary outcome.

### Risk of bias assessment

Hasle 2016, Goldcare 2004, and Hill 2003 showed low risk in most items [4, 13, 15]. In addition, Sullivan 2006 did not describe how they performed their statistical analysis or calculation of the sample size or inclusion criteria, even though all the characteristics are represented in Table 1. The selection bias was reduced in Patja 2006, because patients from all levels of care were included in the Danish database, and its coverage was 99%. Nevertheless, there is a risk of information bias due to patients who migrated and being lost to follow-up. Ehara’s 2011 study was based on an autopsy database, and the data are incomplete in some cases, which can induce information bias, as mentioned by the authors. Though they describe in tables the distribution by age, sex, and tumor type, these have not been adequately described [2, 14, 16] (Table 2).

**Table 2:**
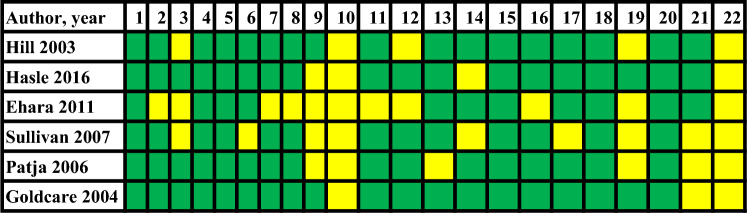
Risk of bias assessment

Fully fulfilled or low risk is represented by green, and not met or high risk is represented by red. Items 1–22 are based on the STROBE checklist available for consultation online at https: www.strobe-statement.org

### Incidence of urological tumors

We identified 42 urological tumors in people with Down's syndrome. There was a total incidence of 0.1%, 95%CI (0.06–0.19), I^2^ 61%.

### Subgroup analysis

**Fig. 2:**
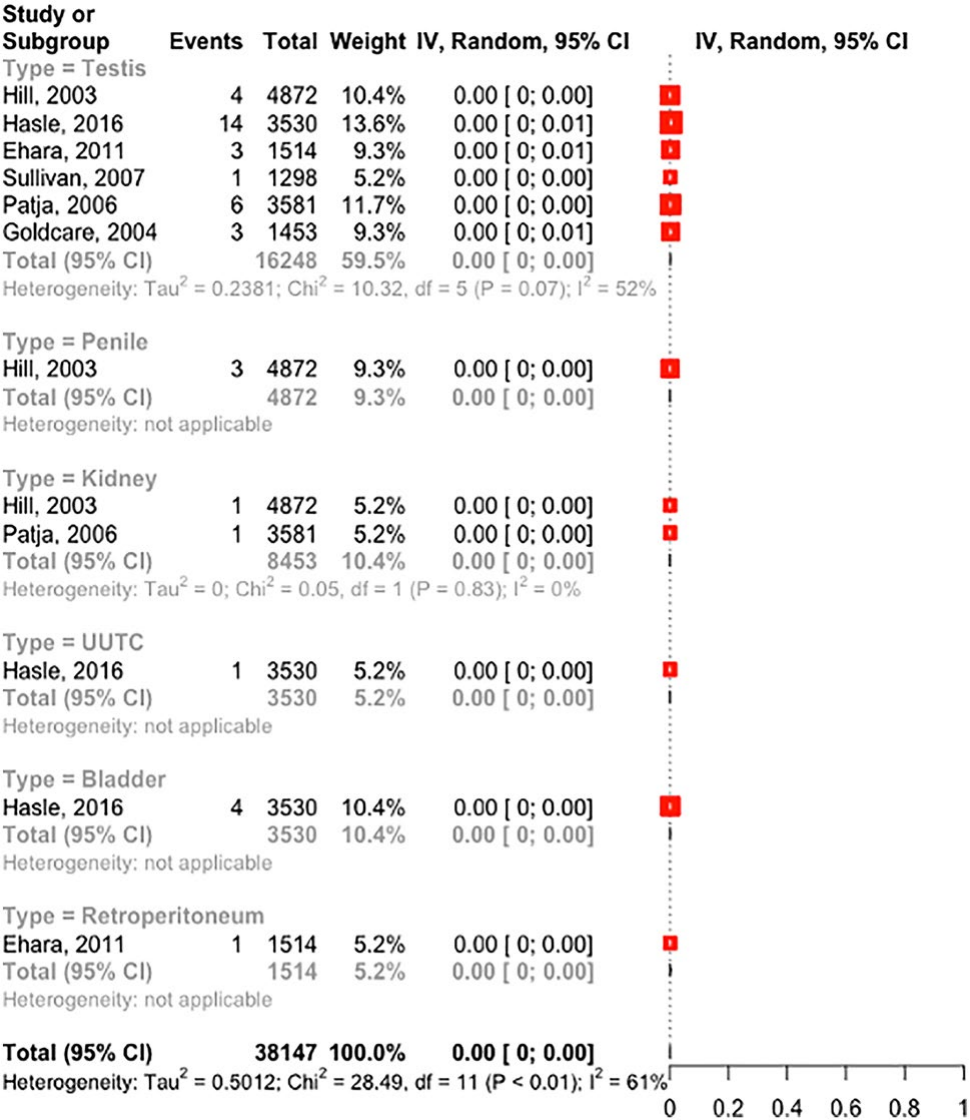
Forrest plot of incidence of tumors in Down’s syndrome

The most common urological tumor was testicular. We found six studies describing 31 events and an overall incidence of 0.19%, 95%CI (0.11–0.33), I^2^: 51%. Other studies reported a very low incidence of kidney, penile, upper urinary tract, bladder, and retroperitoneal tumors (Fig. 2).

### Sensitivity analysis

We did not find any difference when performing the sensitivity analysis.

## Discussion

In contrast to the general population, an estimated rate of new cases of testicular cancer of 6.0 per 100,000 men per year was observed in the USA (SEER). Approximately, 0.4% of men will be diagnosed with testicular cancer during their lifetime, and almost 75% of these cases will occur between 20 and 44 years of age. Furthermore, less than 20% of testicular cancer cases will appear after age 45 [17]. Likewise, in our findings, testicular cancer was present between 20 and 45 years, and none after this age.

Our study has found 31 cases of testicular cancer among 8720 males (16,248 individuals), representing a lifetime incidence of 355 new cases for 100,000 patients with Down syndrome. These numbers mean that 1 in every 281 males with Down syndrome was diagnosed with testicular cancer. Ehara et al. [14], studying autopsies, found 0.36% of testicular tumors in the Japanese population. Nonetheless, other primary studies based on national databases estimated a total lifetime risk of a patient with Down’s syndrome developing testicular cancer of around 0.355%. Nonetheless**,** when reviewing the standardized incidence rates, there were higher values: Patja et al. found a testicular tumor incidence of 4.8, Chicoine et al. described 2.63 times more risk for the Down’s syndrome population, and Hasle et al. found that these tumors were three times more than that expected in the general population [2–4].

According to the SEER database, 0.4% is the estimated lifetime risk of developing testicular cancer for the general population, and this is very similar for patients with Down syndrome in our results. In the same way, regarding the histological subtypes in testicular tumors, seminoma is the most common (58.38%), followed by non-seminoma (40.7%) [17]. Accordingly, we found a similar distribution of testicular tumor subtypes in patients with Down syndrome.

In our study, non-testicular urological tumors were present much less frequently in patients with Down syndrome. However, there is no previous meta-analysis in this regard. The standardized incidence ratio for kidney cancer of 0.6 is mentioned in some studies, such as those by Hill et al. or Patja et al. [2, 13]. In addition, Hesle et al. showed SIR of 2.36 for pelvis and ureter tumors, while SIR was 1.0 for bladder tumors [4]. Based on SEER, kidney cancer incidence was 16.4 per 100,000, and approximately 1.7% of the population will be diagnosed with this tumor sometime during their lifetime.

Regarding non-testicular urological tumors, we found incidences as low as 0.02% in kidney cancer or 0.03% in upper-urothelial tract tumors. It was also lower than that in the general population. Comparing the age of onset of patients, it was also lower than that in the general population, perhaps related to a shorter life expectancy.

### Strengths and limitations

We carried out a very well-designed systematic review of a specific population. We included studies worldwide, pooling general and subtype information on urological tumors.

As a limitation, we found a high heterogeneity and a lack of information regarding non-testicular tumors. However, these findings were consistent with the literature. Other limitations were that follow-up time was not possible to be determined. Likewise, information was generally based on national databases, which might under-report cancer in this population and can not only lead to information bias, but also conduce a selection bias because this population has perhaps stricter follow-up than the general population. There may be stricter follow-up regarding pathologies, especially at younger ages [17]. However, the incidence of urological tumors is lower for the population with Down syndrome than for the general population. However, a finding like that described for a similar population was found for testicular cancer. Therefore it cannot be concluded that the selection risk impacted our data, since both incidences would have had an upward behavior.

## Conclusions

The Down syndrome population had a very low incidence of urological tumors. Testis tumor was the most frequently described in all cohorts, but similar to the general population. Urologists must carefully examine patients with this condition to prevent missing this tumor.

## Appendix 1. Search strategy

### Medline (Ovid)

(Exp urologic neoplasms or (urologic* neoplasm*).mp or (urologic* tumor*).mp or (urologic* cancer*).mp or (urinary tract neoplasm*).mp or (urinary tract tumor*).mp or (urinary tract cancer*).mp or exp kidney neoplasms or (kidney neoplasm*).mp or (kidney tumor*).mp or (kidney cancer*).mp or (renal neoplasm*).mp or (renal tumor*).mp or (renal cancer*).mp or exp ureteral neoplasms or (ureter* neoplasm*).mp or (ureter* tumor*).mp or (ureter* cancer*).mp or exp urethral neoplasms or (urethral neoplasm*).mp or (urethral tumor*).mp or (urethral cancer*).mp or exp urinary bladder neoplasms or (bladder neoplasm*).mp or (bladder tumor*).mp or (bladder cancer*).mp or exp prostatic neoplasms or (prostat* neoplasm*).mp or (prostat* tumor*).mp or (prostat* cancer*).mp or exp penile neoplasms or (peni* neoplasm*).mp or (peni* tumor*).mp or (peni* cancer*).mp or exp testicular neoplasms or (testi* neoplasm*).mp or (testi* tumor*).mp or (testi* cancer*).mp) AND (Exp down syndrome or (down syndrome).mp or mongolism.mp or (trisomy G).mp or (trisomy 21).mp).

### Central (Ovid)

(Exp urologic neoplasms or (urologic* neoplasm*).mp or (urologic* tumor*).mp or (urologic* cancer*).mp or (urinary tract neoplasm*).mp or (urinary tract tumor*).mp or (urinary tract cancer*).mp or exp kidney neoplasms or (kidney neoplasm*).mp or (kidney tumor*).mp or (kidney cancer*).mp or (renal neoplasm*).mp or (renal tumor*).mp or (renal cancer*).mp or exp ureteral neoplasms or (ureter* neoplasm*).mp or (ureter* tumor*).mp or (ureter* cancer*).mp or exp urethral neoplasms or (urethral neoplasm*).mp or (urethral tumor*).mp or (urethral cancer*).mp or exp urinary bladder neoplasms or (bladder neoplasm*).mp or (bladder tumor*).mp or (bladder cancer*).mp or exp prostatic neoplasms or (prostat* neoplasm*).mp or (prostat* tumor*).mp or (prostat* cancer*).mp or exp penile neoplasms or (peni* neoplasm*).mp or (peni* tumor*).mp or (peni* cancer*).mp or exp testicular neoplasms or (testi* neoplasm*).mp or (testi* tumor*).mp or (testi* cancer*).mp) AND (Exp down syndrome or (down syndrome).mp or mongolism.mp or (trisomy G).mp or (trisomy 21).mp).

### Embase

(('urinary tract tumor'/exp or 'urologic* neoplasm*':ti,ab or 'urologic* tumor*':ti,ab or 'urologic* cancer*':ti,ab or 'urinary tract neoplasm*':ti,ab or 'urinary tract tumor*':ti,ab or 'urinary tract cancer*':ti,ab or 'kidney tumor'/exp or 'kidney neoplasm*':ti,ab or 'kidney tumor*':ti,ab or 'kidney cancer*':ti,ab or 'renal neoplasm*':ti,ab or 'renal tumor*':ti,ab or 'renal cancer*':ti,ab or 'ureter tumor'/exp or 'ureter* neoplasm*':ti,ab or 'ureter* tumor*':ti,ab or 'ureter* cancer*':ti,ab or 'urethra tumor'/exp or 'urethral neoplasm*':ti,ab or 'urethral tumor*':ti,ab or 'urethral cancer*':ti,ab or 'bladder tumor'/exp or 'bladder neoplasm*':ti,ab or 'bladder tumor*':ti,ab or 'bladder cancer*':ti,ab or 'prostate tumor'/exp or 'prostat* neoplasm*':ti,ab or 'prostat* tumor*':ti,ab or 'prostat* cancer*':ti,ab or 'penis tumor'/exp or 'peni* neoplasm*':ti,ab or 'peni* tumor*':ti,ab or 'peni* cancer*':ti,ab or 'testis tumor'/exp or 'testi* neoplasm*':ti,ab or 'testi* tumor*':ti,ab or 'testi* cancer*':ti,ab) AND ('Down syndrome'/exp or 'down syndrome':ti,ab or mongolism:ti,ab or 'trisomy G':ti,ab or 'trisomy 21':ti,ab)) AND [embase]/lim.
